# Radiation-free cochlear implant position estimation in pediatric patients using impedance telemetry

**DOI:** 10.1186/s12887-025-06242-y

**Published:** 2025-10-11

**Authors:** Julia Veloso de Oliveira, Enrike Rosenkranz, Stephan Schraivogel, Nora Magdalena Weiss, Marco Caversaccio, Dennis Hedderich, Wilhelm Wimmer

**Affiliations:** 1 Department of Otorhinolaryngology, Klinikum rechts der Isar, Technical University of Munich, Munich, Germany; 2 Department of Neuroradiology, Klinikum rechts der Isar, Technical University of Munich, Munich, Germany; 3 ARTORG Center for Biomedical Engineering Research, University of Bern, Bern, Switzerland; 4 Department of Otorhinolaryngology, Bern University Hospital, Bern, Switzerland

**Keywords:** Objective measures, Radiation exposure, Cochlear anatomy, Follow-up, Electrode position, Anatomy-based fitting

## Abstract

**Background:**

Cochlear implants (CIs) play a crucial role in providing children with profound hearing loss the ability for auditory perception and spoken language development. Following implantation, patients typically undergo a computed tomography (CT) or X-ray examination to assess electrode positions in the cochlea. Besides economic factors, this imposes radiation risks on patients, particularly for younger patients who are more sensitive to its harmful effects.

**Objective:**

This study aims to evaluate the performance of an impedance telemetry-based estimation algorithm for assessing postoperative CI placement. While the algorithm has been validated in an adult cohort, this research explores its applicability in pediatric patients.

**Materials and methods:**

The insertion depth estimation algorithm was validated on a dataset of 59 pediatric cases and evaluated using different classification metrics. Impedance telemetry data was combined with demographic data and morphological parameters of the cochlea and used as input data for the algorithm. The algorithm predictions were compared with ground truth labels derived from X-ray and CT scans.

**Results:**

The algorithm demonstrated a root mean squared error (RMSE) of 1.53mm for insertion depth prediction, compared to 1.01mm in the original report. In terms of classification, the algorithm successfully detected all cases with partially inserted electrodes, although three cases were detected as false positives.

**Conclusion:**

The results highlight the algorithm’s potential for clinical decision support in assessing post-implantation outcomes, monitoring electrode migration, and detecting extracochlear electrodes. A larger dataset with more partial insertion cases would be required for additional training and validation of the algorithm. Assessing electrode placement through impedance measurements would eliminate the need for additional radiography, reducing radiation exposure for future patients while also saving the clinic significant cost and time.

**Supplementary Information:**

The online version contains supplementary material available at 10.1186/s12887-025-06242-y.

## Introduction

CIs have revolutionized the treatment of hearing impairment and significantly improved the quality of life for those affected [1]. These devices are particularly life-changing for children born with profound or complete hearing loss, enabling them to develop language skills and participate in mainstream education [2]. The full and stable placement of the CI electrode array into the cochlea is essential to enable electrical stimulation of the cochlear nerve and ensure successful clinical outcomes.

Typically, post-operative imaging techniques such as CT, cone-beam CT, or X-ray projections are used to verify electrode placement in the cochlea [3, 4]. Although radiographic imaging is effective, it has multiple drawbacks, such as ionizing radiation exposure and the necessity for specialized staff and equipment. In particular, children are found to have a higher likelihood of developing leukemia and brain tumors following CT scans [5, 6]. A recent large-scale study on the effects from CT radiation doses received at a young age reported an expected 1.4 cases of hematological malignancies for every 10,000 examined children [7]. Moreover, an increased risk of radiation-induced solid cancer in pediatric cohorts and specially young girls was reported. For cranial CTs, the risk of leukemia was most elevated in children below 5 years, affecting 1.9 patients for every 10,000 cases [8].

Another important aspect concerns the costs and risks associated with the use of sedation or anesthesia during radiologic procedures. This is a necessary step to ensure body stillness during examination, since young patients cannot remain motionless for the entire duration of the examination [9]. Although serious complications due to sedation and/or anesthesia such as cardiac arrest and seizure are rare, other potentially harmful adverse events such as respiratory depression, hypoxia, and vomiting can occur [10]. From a financial perspective, the use of sedation or anesthesia also increases the overall costs, not only due to the medication acquisition but also due to the longer clinic visits and the associated personnel costs [11].

Recent studies have demonstrated the potential of impedance telemetry for postoperative CI assessment without the need for radiographic imaging. In clinical settings, these measurements are routinely obtained using the implant’s reverse telemetry capabilities to verify device integrity and identify faulty contacts. Beyond this standard use, impedance data, including the voltage matrix, also referred to as the transimpedance matrix in the literature, has been used to detect extracochlear electrodes [12, 13], electrode array tip fold-over [14–16], and scalar translocation [17]. Furthermore, impedance-based methods could enable the estimation of the electrode distance to the cochlear walls [18–20] and the electrode array linear insertion depth [21, 22]. Finally, impedance recordings can potentially provide more information than clinical imaging currently does, such as on intracochlear bleeding [23] and postoperative fibrotic tissue formation [24–26].

Schraivogel et al. recently proposed an impedance-based method to estimate CI electrode array insertion depth without the need for postoperative imaging [27]. The novel approach uses a machine-learning algorithm to predict the insertion depth in millimeters based on features extracted from the voltage matrix and cochlear dimensions measured from preoperative CT images. So far, validation of the best performing algorithm has been carried out in adult cohorts, with ground truth electrode position labels derived from CT images [22, 27]. Since the inner ear is fully developed before birth [28] and the method is particularly sensitive to the macro-anatomy of the cochlea [27], the algorithm is expected to be applicable to pediatric cohorts as well. For a fully inserted electrode array, the insertion depth can be predicted with an average accuracy of around 1 millimeter [27], which is sufficient to make a clinically relevant statement.

The aim of this study is to evaluate the performance of a radiation-free, impedance-based algorithm for estimating CI insertion depth in pediatric patients. To this end, the algorithm was validated on an independent dataset from a different clinic. Our focus is on the classification performance in determining whether the electrode array is fully or partially inserted into the cochlea. The algorithm could potentially serve as a decision support tool to verify whether the electrode array was successfully inserted, thereby assisting clinicians in determining the need for further diagnostic steps or intervention.

## Materials and methods

### Study design and demographics

To evaluate the classification performance, we performed a retrospective analysis of pediatric cochlear implant cases at our clinic in Munich from 2007 to 2024. The study protocol was approved by the local institutional review board (ID 2023-367-S-SR). We selected cases involving patients who were up to 6 years old at the time of implantation, and excluded those with cochlear malformations. In the previous study, the insertion depth estimation algorithm focused on adult CI recipients [27]. Consequently, young children were underrepresented despite being a key cohort. This study specifically addresses the pediatric population, particularly around the age of one to two years, which is the most common age for cochlear implantation in our clinic. This developmental stage is also characterized by significant skull growth, which enabled us to test whether these anatomical changes influence the accuracy of our method.

The analysis was restricted to patients who received a MED-EL implant with either Standard, FLEX$^{\textrm{28}}$ or FLEX$^{\textrm{soft}}$ arrays. The FLEX$^{\textrm{28}}$ array, originally used to train the estimation algorithm, is slightly shorter (28mm) than the Standard and FLEX$^{\textrm{soft}}$ arrays (31.5mm). The Standard array contains 12 double-banded electrodes, while the arrays from the FLEX series feature five single-banded electrodes in the apical section and seven double-banded electrodes toward the base. In addition, the algorithm required a complete set of preoperative CT images, postoperative CT or X-ray images and impedance telemetry data. As in the previous study [27], cases with more than 100 days between imaging and telemetry were excluded; in our cohort, a maximum of 38 days was observed.

Altogether, we included 41 patients, of whom 24 received a unilateral CI and 17 received a bilateral CI, with a total of 58 implanted ears (32 left, 26 right). One patient required revision surgery approximately two years after implantation due to electrode array migration [29]. Data collected shortly before the revision were also included in the analysis, resulting in a total of 59 cases in 41 patients (15 female, 26 male) with a mean age at implantation of 2.1 years. No additional cases of explantation or re-implantation were included in the dataset. The etiology of hearing loss remained unidentified in the majority of patients (34). However, certain risk factors were identified, including premature birth (3 patients) and a family history of deafness (2 patients). Two cases were associated with Waardenburg syndrome, one with Aymé-Gripp syndrome [29], two with other genetic conditions, one with cytomegalovirus (CMV) infection, and one with meningitis. A comprehensive overview of the study cohort is provided in Supplementary Table 1.

### Impedance telemetry data

Depending on the data availability, either intraoperative (49 cases) or postoperative (10 cases) CI impedance recordings were considered for the analysis. Intraoperative measurements were conducted after the insertion of the electrode array, while postoperative recordings were performed during follow-up clinic visits. Electrode impedances typically increase postoperatively as a result of foreign body response and fibrous tissue development [24, 30–33]. To accommodate such changes, the estimation model was trained on both intraoperative and postoperative recordings [27]. A single impedance recording per case was considered in the analysis.

**Fig. 1:**
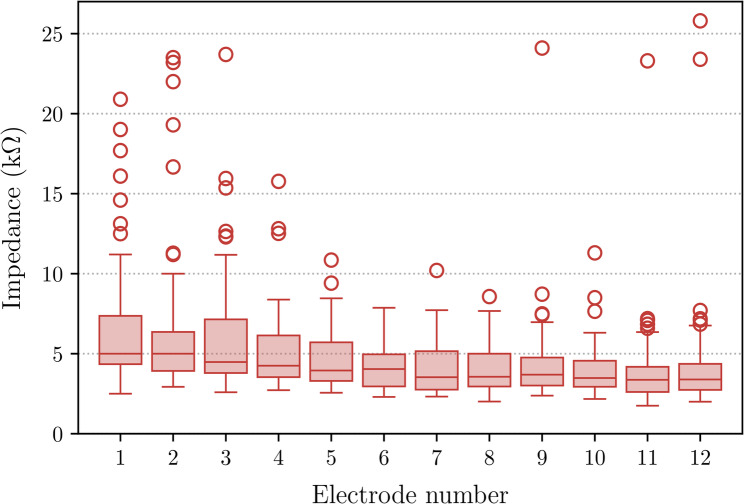
Boxplot of electrode impedances for each channel. Outliers are marked as circles. Electrodes are numbered from apical (1) to basal (12)

All recordings were performed with the manufacturer’s clinical software (MAESTRO 9, MED-EL) using the standard Impedance Field Telemetry (IFT) protocol. Herein, a biphasic charge-balanced stimulus with leading cathodic phase (amplitude and pulse width of approximately 300 µA and 24 µs respectively) is generated by the stimulating electrode contact. The voltage difference between a given electrode and the reference electrode located on the implant body is sampled at the trailing edge of the anodic stimulation pulse [34]. This procedure is repeated for each combination of electrode pairs used as stimulating and recording contacts, resulting in the so-called voltage matrix. In this context, impedances (in $\Omega$) are defined as the ratio of the recorded voltage (in V) to the stimulation current (in A). No short circuits or open circuits were present in the dataset. Figure 1 shows a boxplot of electrode impedances measured using the same electrode for both stimulation and recording. The boxplot reveals a total of 47 outliers, with 15 originating from intraoperative and 32 from postoperative measurements.

### Radiographic assessment

Both preoperative and postoperative radiologic images were included in the data analysis. Cochlear dimensions were extracted from preoperative images and fed to the machine learning algorithm. The electrode array position, estimated from postoperative images, served as ground truth labels for evaluating the algorithm’s performance.

#### Preoperative: anatomical parameters

**Fig. 2:**
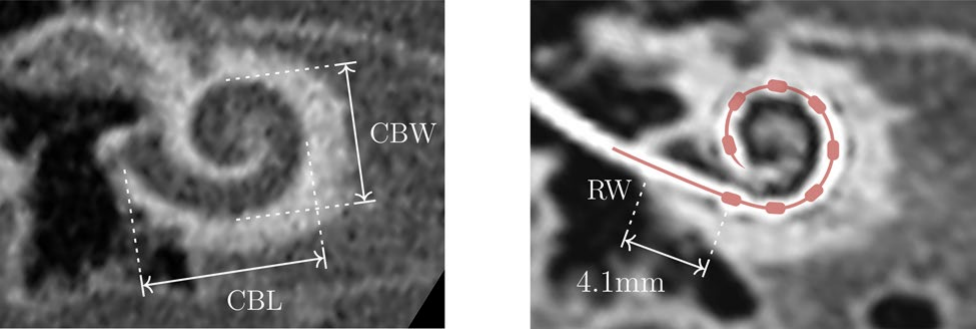
Example of a preoperative (left) and a postoperative (right) CT scan. From the preoperative image, the cochlear base length (CBL) and width (CBW) can be estimated in millimeter. After the implantation, the insertion depth of the electrode array can be assessed by measuring the distance between the round window (RW) and the most basal electrode. In this example, the insertion depth corresponds to 4.1 mm. For visual simplicity, only the eight most basal electrodes are annotated in the postoperative scan

As part of the standard clinical protocol for CI candidacy, every patient undergoes a CT imaging examination to exclude cochlear malformation or ossification [35]. Using the preoperative CT scans, each cochlea was assessed by an expert to obtain its cochlear base length and width (Fig. 2, left) [36]. These anatomical parameters served as input to the algorithm for insertion depth estimation. For the three cases lacking preoperative CT scans, cochlear metrics were derived either from existing magnet resonance imaging data or substituted with the average values calculated from the rest of the cohort.

#### Postoperative: electrode insertion depth

**Fig. 3:**
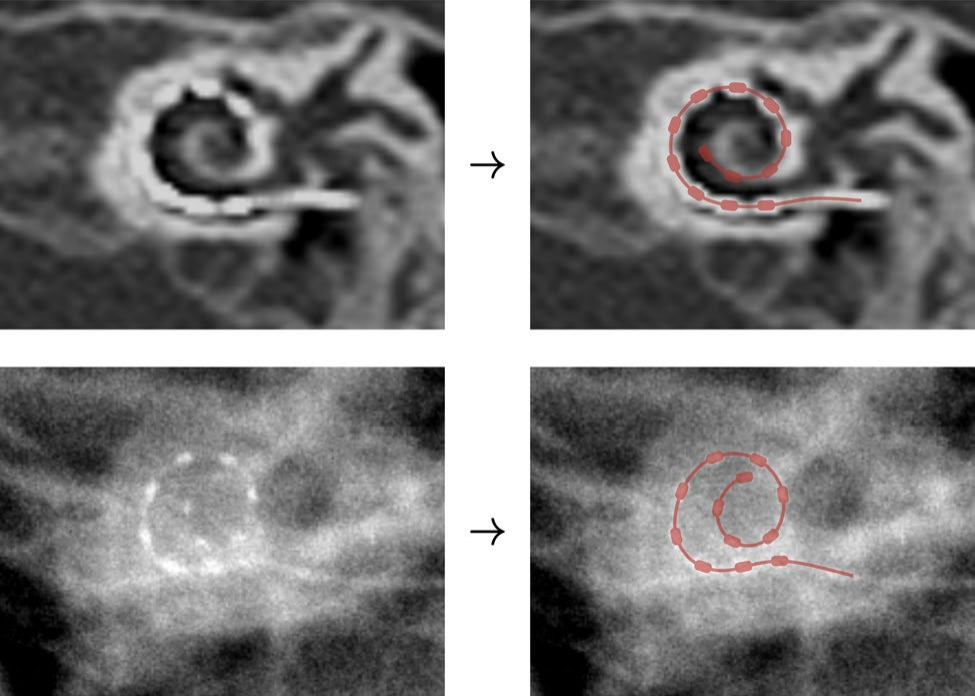
Postoperative CT scan (top) and X-ray (bottom) for the assessment of the electrode array position. The twelve electrode contacts are highlighted in red

As part of the clinical routine, an X-ray image was obtained for all patients within 5 days post-implantation using either Xu’s or Stenver’s view [3, 37]. Only in cases with complications during the follow-up (e.g., suspected electrode migration, vertigo, suspected mastoiditis, sudden non-acceptance of the audio processor, and fall incidents), an additional CT examination was performed. The linear insertion depth of the most basal electrode in relation to the round window was determined from either CT or X-ray images (Fig. 2, right) and was used as the ground truth label. In the revised case, labels were extracted from the latest CT scan before the revision surgery (approximately 4 months). Two examples of postoperative X-ray and CT images are given in Fig. 3.

### Impedance-based insertion depth estimation

The algorithm detailed in [27] was selected for predicting electrode array linear insertion depth. In summary, a decision-tree based model “Extra Trees” was identified in the original study as the best performing model to predict the insertion depth in a dataset of 118 cochlear implantation cases. The model takes as input voltage matrices acquired through standard CI impedance telemetry measurements, along with cochlear dimensions extracted from preoperative radiographic imaging. It outputs an estimate of the linear insertion depth (in mm) of the most basal electrode relative to the round window. The Extra Trees model achieved a mean absolute error (MAE) of 0.8mm ± 0.6mm (mean ± standard deviation) for predicting the linear insertion depth of the most basal electrode using leave-one-out cross-validation. The same algorithm was evaluated on an independent hold-out dataset of 13 cases and achieved a MAE of 1.37mm ± 0.97mm and a RMSE of 1.67mm. The novel algorithm was also applicable to cases with partially inserted electrode arrays.

The current evaluation focuses on the clinical applicability of the algorithm in a pediatric cohort. The goal is to provide an initial statement about the full or partial electrode array insertion to define the next steps in the CI rehabilitation. A partial insertion can be identified if the algorithm output is negative, meaning the most basal electrode is located outside the cochlea.

### Statistical analysis

In order to assess the algorithm’s prediction accuracy (in millimeters), the mean absolute error (MAE) and root mean squared error (RMSE) with respect to the ground truth were calculated. For classification performance analysis, we utilized a confusion matrix and measured sensitivity, specificity, and overall accuracy compared to manual annotations.

## Results

### Ground truth labels

A full insertion (non-negative linear insertion depth) could be confirmed in 56 out of 59 cases, with absolute insertion depth values ranging from 0 mm to 5.1mm. Of the three partial insertion cases, two extracochlear electrodes were observed in two cases (–5mm and –6.2mm) and a single extracochlear electrode was identified in one case (–1.4mm). Detailed information on all cases can be found in Supplementary Table 1.

### Algorithm performance

**Fig. 4:**
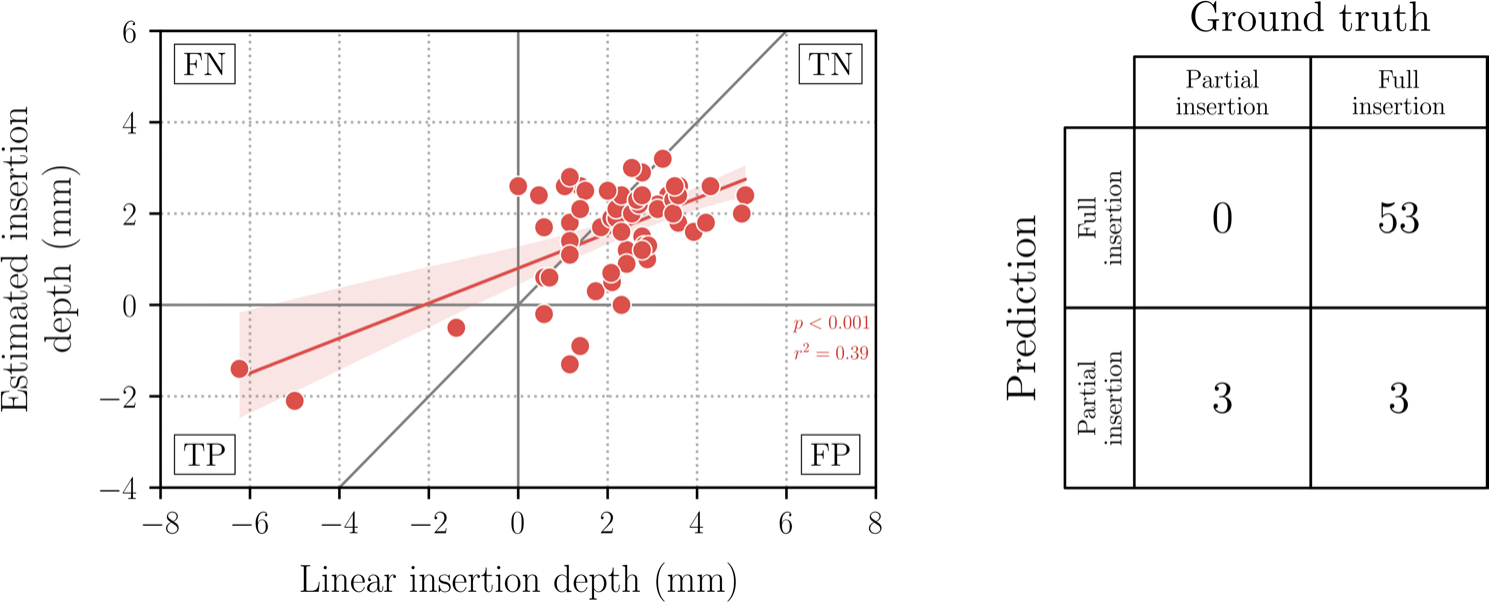
Left: Scatter plot of the ground truth insertion depth and the estimated insertion depth using the machine learning algorithm. The red line represents the regression fit of ground truth values and predictions. For comparison, a perfect line fit (in gray) is included in the diagram. FP: False positive. TP: True positive. TN: True negative. FN: False negative. Right: Confusion matrix summarizing the output of the algorithm compared to the manual annotations

The predicted electrode array insertion depth was compared to the manually extracted ground truth labels for all 59 cases. Figure 4 shows a scatter plot (left) and a confusion matrix (right) of the prediction outcomes. The prediction error ranged from –3mm to 4.8mm with an MAE of 1.2mm ± 0.9mm (mean ± standard deviation). An RMSE of 1.53mm was observed. In terms of classification metrics, extracochlear electrodes were identified with an accuracy of 95%, a sensitivity of 100% and a specificity of 95%. Table 1 provides a summary of these results, along with those from Schraivogel et al. [27].

**Table 1:** Best algorithm performance in the original study compared to the pediatric dataset presented in this study. MAE: Mean absolute error. RMSE: Root mean squared error

	Schraivogel et al. [27]	Pediatric cohort
MAE (mm)	1.4 ± 1.0	1.2 ± 0.9
RMSE (mm)	1.67	1.53
[min, max]_error_ (mm)	[−3.1, 2.9]	[−3.0, 4.8]
Accuracy	85%	95%
Specificity	82%	95%
Sensitivity	100%	100%

## Discussion

This work assessed the performance of a machine learning algorithm in predicting electrode array insertion depth in a pediatric dataset. Impedance telemetry recordings as well as CT and X-ray images were collected for the evaluation. The algorithm’s predictions were compared with ground truth labels annotated by an expert in postoperative radiological images. The findings of this study are comparable to the algorithm’s performance on the hold-out dataset reported by Schraivogel et al. [27]. The MAE was slightly lower in our cohort, with 1.2mm ± 0.9mm compared to 1.4mm ± 1.0mm. Similarly, the RMSE showed a modest improvement, decreasing from 1.67mm in the original study to 1.53mm in our cohort. The largest absolute prediction error (4.8mm) was observed in a patient with significant electrode array migration (–6.2mm, subject S28). In this case, the impedance recording was performed intraoperatively, and channel 9 exhibited an abnormally high value of 24 k$\Omega$. This may have been caused by an air bubble that had not yet dissolved at the time of measurement. The poor performance of the algorithm for this subject could be attributed to the atypical impedance pattern. Additionally, the algorithm’s training data did not include any cases with an electrode array insertion depth smaller than –5mm [27].

Both classification accuracy and specificity rose to 95% in the present study. In addition, the perfect sensitivity score indicates that the algorithm successfully identified all cases with extracochlear electrodes, even though not all detected cases were true partial insertions. Out of the three false positives, two correspond to FLEX$^{28}$ electrode arrays and one to a Standard array. These false positives could be a result of electrode migration between the impedance measurement and the imaging examination. Ideally, both assessments should be conducted on the same day; however, this is not always feasible due to clinical workflow constraints. It is important to note that the algorithm was previously trained on an independent dataset from a different clinic and used in this study without further adjustments. Therefore, the present study provides a good estimate of the algorithm’s generalization performance in clinical practice in pediatric patients.

There’s an important distinction to be made between linear insertion depth and angular insertion depth. The linear insertion depth, as estimated by the algorithm, refers to the physical length of the electrode array that has been inserted into the cochlea, usually measured in millimeters from the round window or cochleostomy. It can be estimated visually by the surgeon during the implantation. If the cochlear duct length is known, the linear insertion depth can be used to map frequencies using the Greenwood function [38]. In contrast, the angular insertion depth describes the angular position with respect to the cylindrical coordinate system, measured in degrees from the round window (0°). The angular insertion depth allows for more standardized comparison between patients because it accounts for differences in cochlear size and shape [39, 40]. Converting the estimated linear insertion depth to an angular insertion depth is feasible, for example using the method introduced in [21]. Although the algorithm estimates the linear insertion depth of the most basal contact, both linear and angular insertion depths for all electrode contacts can be derived using the known geometry of the electrode array and the cochlear dimensions [4, 41].

To estimate the linear insertion depth, we primarily relied on postoperative X-rays and used CT scans when available. X-ray imaging is widely used for assessment of insertion depth, providing estimates that correlate well with those from CT scans [42–45]. When higher detail is required, conventional CT or cone-beam CT offers improved spatial resolution and enables three-dimensional visualization, although at the cost of higher radiation exposure [3, 46]. Annotations from both conventional CT and cone-beam CT have been used in previous studies [21, 22, 47, 48] and could similarly serve as reference data for validating the estimation algorithm.

The algorithm’s capability to identify partial insertion of electrodes was assessed because of its potential use in clinical settings. The high sensitivity qualifies the algorithm as a potential clinical decision-support tool that can provide additional evidence for distinguishing between full and partial electrode array insertion. This is particularly relevant, as partial insertions can occur without the elevated basal impedances typically associated with extra-cochlear electrodes [49]. In this context, the algorithm could serve as an efficient, inexpensive, fast indicator of extracochlear electrodes, potentially eliminating the need for postoperative imaging. Since the algorithm is based on telemetry data recorded with biphasic pulses, the most common stimulus shape in clinical settings, it could be integrated into routine care without requiring changes to stimulation protocols.

Furthermore, the algorithm can be utilized as a preliminary screening tool for detecting electrode array migration, which may be supplemented by a radiological exam if necessary [50]. The clinical application of the algorithm has been recently reported in an electrode migration case [29]. The electrode array, initially fully inserted, gradually migrated out of the cochlea over a period of months. In retrospect, the migration of the most basal electrode, and later the second most basal electrode, was successfully tracked by the prediction algorithm.

The current study presents some limitations. Our dataset included only three cases of partial electrode array insertion, which limits the significance of the findings. A larger sample size would be necessary to draw more general conclusions about the algorithm’s ability to detect extracochlear electrodes and its clinical significance. Additionally, only 17 out of 59 cases were implanted with a FLEX$^{\textrm{28}}$ array, which is the only electrode design the current algorithm was originally trained on. Future models should be trained also on other electrode array types to reduce estimation errors in these cases.

## Conclusion

The goal of this work was to validate a previously trained machine learning algorithm on an independent cohort of pediatric cochlear implantation cases. The dataset included 59 cases of 41 patients and contained both partial and full electrode array insertions. Ground truth labels were extracted from X-ray and CT images and compared to the algorithm’s output. Results suggest that the algorithm can be applied clinically to detect extracochlear electrodes, potentially eliminating the need for postoperative imaging. Further applications include, for example, the monitoring of electrode migration in the follow-up. The findings of this study could be further improved by including more partial insertion cases in the validation set.

## Supplementary Information

Supplementary Material 1.

## Data Availability

The data that support the findings of this study are not openly available due to reasons of sensitivity and are available from the corresponding author upon reasonable request.

## References

[1] Wilson BS, Dorman MF. Cochlear implants: a remarkable past and a brilliant future. Hear Res. 2008;242(1–2):3–21. 18616994 10.1016/j.heares.2008.06.005 PMC3707130

[2] Niparko JK, Tobey EA, Thal DJ, Eisenberg LS, Wang NY, Quittner AL, et al. Spoken language development in children following cochlear implantation. JAMA. 2010;303(15):1498–506. 20407059 10.1001/jama.2010.451 PMC3073449

[3] Wimmer W, Bell B, Huth ME, Weisstanner C, Gerber N, Kompis M, et al. Cone beam and micro-computed tomography validation of manual array insertion for minimally invasive cochlear implantation. Audiol Neurotol. 2014;19(1):22–30. 10.1159/000356165 24280962

[4] Rathgeb C, Demattè M, Yacoub A, Anschuetz L, Wagner F, Mantokoudis G, et al. Clinical applicability of a preoperative angular insertion depth prediction method for cochlear implantation. Otol Neurotol. 2019;40(8):1011–7. 31419213 10.1097/MAO.0000000000002304

[5] Pearce MS, Salotti JA, Little MP, McHugh K, Lee C, Kim KP, et al. Radiation exposure from ct scans in childhood and subsequent risk of leukaemia and brain tumours: a retrospective cohort study. Lancet. 2012;380(9840):499–505. 10.1016/s0140-6736(12)60815-0. 22681860 10.1016/S0140-6736(12)60815-0 PMC3418594

[6] King MA, Kanal KM, Relyea-Chew A, Bittles M, Vavilala MS, Hollingworth W. Radiation exposure from pediatric head ct: a bi-institutional study. Pediatr Radiol. 2009;39(10):1059–65. 10.1007/s00247-009-1327-1. 19554322 10.1007/s00247-009-1327-1

[7] Bosch de Basea Gomez M, Thierry-Chef I, Harbron R, Hauptmann M, Byrnes G, Bernier MO, et al. Risk of hematological malignancies from CT radiation exposure in children, adolescents and young adults. Nat Med. 2023;29(12):3111–9. 10.1038/s41591-023-02620-0. 10.1038/s41591-023-02620-0 PMC10719096 37946058

[8] Miglioretti DL, Johnson E, Williams A, Greenlee RT, Weinmann S, Solberg LI, et al. The use of computed tomography in pediatrics and the associated radiation exposure and estimated cancer risk. JAMA Pediatr. 2013;167(8):700–7. 23754213 10.1001/jamapediatrics.2013.311 PMC3936795

[9] Erondu OF. Challenges and peculiarities of paediatric imaging. Med Imaging Clin Pract. 2013;23(23–35):156.

[10] Cravero JP, Blike GT, Beach M, Gallagher SM, Hertzog JH, Havidich JE, et al. Incidence and nature of adverse events during pediatric sedation/anesthesia for procedures outside the operating room: report from the pediatric sedation research consortium. Pediatrics. 2006;118(3):1087–96. 10.1542/peds.2006-0313. 16951002 10.1542/peds.2006-0313

[11] Vanderby SA, Babyn PS, Carter MW, Jewell SM, McKeever PD. Effect of anesthesia and sedation on pediatric mr imaging patient flow. Radiology. 2010;256(1):229–37. 10.1148/radiol.10091124. 20505061 10.1148/radiol.10091124

[12] Rijk SR, Tam YC, Carlyon RP. Detection of extracochlear electrodes in cochlear implants with electric field imaging/transimpedance measurements: a human cadaver study. Ear Hear. 2020;41(5):1196–207. 10.1097/aud.0000000000000837. 31923041 10.1097/AUD.0000000000000837 PMC7115972

[13] Rijk SR, Hammond-Kenny A, Tam YC, Eitutis ST, Garcia C, Carlyon RP, et al. Detection of extracochlear electrodes using stimulation-current- induced non-stimulating electrode voltage recordings with different electrode designs. Otol Neurotol. 2022;43(5):e548–57. 35617005 10.1097/MAO.0000000000003512

[14] Klabbers TM, Huinck WJ, Mylanus EAM. Comparison between transimpedance matrix (tim) measurement and x-ray fluoroscopy for intraoperative electrode array tip fold-over detection. Otol Neurotology. 2021;42(10):e1457–63. 10.1097/mao.0000000000003290. 10.1097/MAO.0000000000003290 34238897

[15] Hans S, Arweiler-Harbeck D, Kaster F, Ludwig J, Hagedorn E, Lang S, et al. Transimpedance matrix measurements reliably detect electrode tip fold-over in cochlear implantation. Otol Neurotology. 2021;42(10):e1494–502. 10.1097/mao.0000000000003334. 10.1097/MAO.0000000000003334 34766947

[16] Ramos de Miguel Á, Riol Sancho D, Falcón-González JC, Pavone J, Rodríguez Herrera L, Borkoski Barreiro S, et al. Assessing the placement of the cochlear slim perimodiolar electrode array by trans Impedance Matrix analysis: A temporal bone study. J Clin Med. 2022;11(14):3930. 10.3390/jcm11143930 PMC9317462 35887693

[17] Dong Y, Briaire JJ, Siebrecht M, Stronks HC, Frijns JHM. Detection of translocation of cochlear implant electrode arrays by intracochlear impedance measurements. Ear Hear. 2021;42(5):1397–404. 10.1097/aud.0000000000001033. 33974777 10.1097/AUD.0000000000001033 PMC8378542

[18] Sijgers L, Huber A, Tabibi S, Grosse J, Roosli C, Boyle P, et al. Predicting cochlear implant electrode placement using monopolar, three-point and four-point impedance measurements. IEEE Trans Biomed Eng. 2022;69(8):2533–44. 10.1109/tbme.2022.3150239. 35143392 10.1109/TBME.2022.3150239

[19] Giardina CK, Krause ES, Koka K, Fitzpatrick DC. Impedance measures during in vitro cochlear implantation predict array positioning. IEEE Trans Biomed Eng. 2018;65(2):327–35. 10.1109/tbme.2017.2764881. 29346102 10.1109/TBME.2017.2764881 PMC5929978

[20] Bruns TL, Riojas KE, Labadie RF, Webster RJ Iii. Real-time localization of cochlear-implant electrode arrays using bipolar impedance sensing. IEEE Trans Biomed Eng. 2022;69(2):718–24. 10.1109/TBME.2021.3104104 PMC8918040 34379586

[21] Aebischer P, Meyer S, Caversaccio M, Wimmer W. Intraoperative impedance-based estimation of cochlear implant electrode array insertion depth. IEEE Trans Biomed Eng. 2021;68(2):545–55. 10.1109/tbme.2020.3006934. 32746052 10.1109/TBME.2020.3006934

[22] Schraivogel S, Aebischer P, Wagner F, Weder S, Mantokoudis G, Caversaccio M, et al. Postoperative impedance-based estimation of cochlear implant electrode insertion depth. Ear Hear. 2023;44(6):1379–88. 37157125 10.1097/AUD.0000000000001379 PMC10583924

[23] Bester C, Razmovski T, Collins A, Mejia O, Foghsgaard S, Mitchell-Innes A, et al. Four-point impedance as a biomarker for bleeding during cochlear implantation. Sci Rep. 2020. 10.1038/s41598-019-56253-w. 32066743 10.1038/s41598-019-56253-w PMC7026160

[24] Leblans M, Sismono F, Vanpoucke F, Dinther J, Lerut B, Kuhweide R, et al. Novel impedance measures as biomarker for intracochlear fibrosis. Hear Res. 2022;426:108563. 10.1016/j.heares.2022.108563. 35794046 10.1016/j.heares.2022.108563

[25] Sijgers L, Geys M, Geissler G, Boyle P, Huber A, Pfiffner F. Electrical bioimpedance-based monitoring of intracochlear tissue changes after cochlear implantation. Sensors. 2024;24(23):7570. 10.3390/s24237570. 39686105 10.3390/s24237570 PMC11644215

[26] Buswinka CJ, Colesa DJ, Swiderski DL, Raphael Y, Pfingst BE. Components of impedance in a cochlear implant animal model with TGFβ1-accelerated fibrosis. Hear Res. 2022;426:108638. 10.1016/j.heares.2022.108638. 36368194 10.1016/j.heares.2022.108638 PMC10794021

[27] Schraivogel S, Weder S, Mantokoudis G, Caversaccio M, Wimmer W. Predictive models for radiation-free localization of cochlear implants’ most basal electrode using impedance telemetry. IEEE Trans Biomed Eng. 2025;72(4):1453–64. 10.1109/TBME.2024.3509527. 40030461 10.1109/TBME.2024.3509527

[28] Dahm MC, Shepherd RK, Clark GM. The postnatal growth of the temporal bone and its implications for cochlear implantation in children. Acta Otolaryngol. 1993;113(sup505):4–39. 10.3109/00016489309128539. 8379315

[29] Schraivogel S, Regele S, Weiss NM, Wirth M, Wollenberg B, Caversaccio M, et al. Impedance-based detection of cochlear implant array migration: case report in a child with Aymé-Gripp syndrome. Eur Arch Otorhinolaryngol. 2025. 10.1007/s00405-025-09397-7. 40301235 10.1007/s00405-025-09397-7 PMC12399727

[30] Foggia MJ, Quevedo RV, Hansen MR. Intracochlear fibrosis and the foreign body response to cochlear implant biomaterials. Laryngoscope Investig Otolaryngol. 2019;4(6):678–83. 10.1002/lio2.329. 31890888 10.1002/lio2.329 PMC6929576

[31] Newbold C, Richardson R, Millard R, Seligman P, Cowan R, Shepherd R. Electrical stimulation causes rapid changes in electrode impedance of cell-covered electrodes. J Neural Eng. 2011;8(3):036029. 10.1088/1741-2560/8/3/036029. 21572219 10.1088/1741-2560/8/3/036029 PMC3147028

[32] Veloso de Oliveira J, Weiss NM, Wimmer W. Comprehensive decomposition of cochlear implant electrode impedances. Hear Res. 2025;466:109348. 10.1016/j.heares.2025.109348. 10.1016/j.heares.2025.109348 40714629

[33] Andonie R, Caversaccio M, Weder S, Wimmer W. Impedance-based tissue response modeling for the prediction of hearing preservation after cochlear implantation. Comput Biol Med. 2025;196:110626. 10.1016/j.compbiomed.2025.110626. 40614514 10.1016/j.compbiomed.2025.110626

[34] Zierhofer CM, Hochmair IJ, Hochmair ES. The advanced combi 40+ cochlear implant. Am J Otol. 1997;18(6 Suppl):S37-8. 9391589

[35] Wimmer W, Soldati FO, Weder S, Vischer M, Mantokoudis G, Caversaccio M, et al. Cochlear base length as predictor for angular insertion depth in incomplete partition type 2 malformations. Int J Pediatr Otorhinolaryngol. 2022;159:111204. 35696773 10.1016/j.ijporl.2022.111204

[36] Anschuetz L, Weder S, Mantokoudis G, Kompis M, Caversaccio M, Wimmer W. Cochlear implant insertion depth prediction: a temporal bone accuracy study. Otol Neurotol. 2018;39(10):e996-1001. 30303947 10.1097/MAO.0000000000002034

[37] Xu J, Xu SA, Cohen LT, Clark GM. Cochlear view: postoperative radiography for cochlear implantation. Am J Otolaryngol. 2000;21(1):49–56. 10651435

[38] Greenwood DD. A cochlear frequency-position function for several species—29 years later. J Acoust Soc Am. 1990;87(6):2592–605. 10.1121/1.399052. 2373794 10.1121/1.399052

[39] Verbist BM, Skinner MW, Cohen LT, Leake PA, James C, Boëx C, et al. Consensus panel on a cochlear coordinate system applicable in histologic, physiologic, and radiologic studies of the human cochlea. Otol Neurotol. 2010;31(5):722–30. 10.1097/mao.0b013e3181d279e0. 20147866 10.1097/MAO.0b013e3181d279e0 PMC2945386

[40] Wimmer W, Vandersteen C, Guevara N, Caversaccio M, Delingette H. Robust Cochlear Modiolar Axis Detection in CT. In: Medical Image Computing and Computer Assisted Intervention – MICCAI 2019. Springer International Publishing; 2019. pp. 3–10. ISSN: 1611-3349. 10.1007/978-3-030-32254-0_1. 10.1007/978-3-030-32254-0_1 PMC6992420 32002521

[41] Anschuetz L, Weder S, Mantokoudis G, Kompis M, Caversaccio M, Wimmer W. Cochlear implant insertion depth prediction: a temporal bone accuracy study. Otol Neurotology. 2018;39(10):e996–1001. 10.1097/mao.0000000000002034. 10.1097/MAO.0000000000002034 30303947

[42] Sokolov M, Zavdy O, Raveh E, Ulanovski D, Attias Y, Hilly O. Assessment of angular insertion-depth of bilateral cochlear implants using plain x-ray scans. Otol Neurotology. 2020;41(10):1363–8. 10.1097/mao.0000000000002830. 10.1097/MAO.0000000000002830 32890291

[43] Fernandes V, Wang Y, Yeung R, Symons S, Lin V. Effectiveness of skull x-ray to determine cochlear implant insertion depth. J Otolaryngol Head Neck Surg. 2018. 10.1186/s40463-018-0304-9. 30176926 10.1186/s40463-018-0304-9 PMC6122652

[44] Alahmadi A, Abdelsamad Y, Hafez A, Hagr A. X-ray guided anatomy-based fitting: the validity of otoplan. PLoS ONE. 2024;19(11):e0313567. 10.1371/journal.pone.0313567. 39546436 10.1371/journal.pone.0313567 PMC11567511

[45] Noble AR, Christianson E, Norton SJ, Ou HC, Phillips GS, Khalatbari H, et al. Reliability of measuring insertion depth in cochlear implanted infants and children using cochlear view radiography. Otolaryngol Head Neck Surg. 2020;163(4):822–8. 10.1177/0194599820921857. 32450736 10.1177/0194599820921857

[46] Lee SY, Han JH, Carandang M, Bae YJ, Choi BY. Simpler and effective radiological evaluations for modiolar proximity of a slim modiolar cochlear implant electrode. Sci Rep. 2020. 10.1038/s41598-020-74738-x. 33077822 10.1038/s41598-020-74738-x PMC7573622

[47] Ramos-de Miguel A, Falcón-González JC, Ramos-Macias A. Analysis of Neural Interface When Using Modiolar Electrode Stimulation. Radiological Evaluation, Trans-Impedance Matrix Analysis and Effect on Listening Effort in Cochlear Implantation. J Clin Med. 2021;10(17):3962. 10.3390/jcm10173962. 10.3390/jcm10173962 PMC8432261 34501410

[48] Lambriks L, van Hoof M, Debruyne J, Janssen M, Hof J, Hellingman K, et al. Toward neural health measurements for cochlear implantation: the relationship among electrode positioning, the electrically evoked action potential, impedances and behavioral stimulation levels. Front Neurol. 2023. 10.3389/fneur.2023.1093265. 36846130 10.3389/fneur.2023.1093265 PMC9948626

[49] Holder JT, Kessler DM, Noble JH, Gifford RH, Labadie RF. Prevalence of extracochlear electrodes: computerized tomography scans, cochlear implant maps, and operative reports. Otol Neurotology. 2018;39(5):e325. 10.1097/mao.0000000000001818. 10.1097/MAO.0000000000001818 PMC7197293 29738386

[50] Rader T, Baumann U, Stöver T, Weissgerber T, Adel Y, Leinung M, et al. Management of cochlear implant electrode migration. Otol Neurotology. 2016;37(9):e341–8. 10.1097/MAO.0000000000001065 27631657

