# Supplementary material for: Radiation-free cochlear implant position estimation in pediatric patients using impedance telemetry
Source: BMC Pediatr. 2025 Oct 11;25:809. doi: 10.1186/s12887-025-06242-y (PMC12514828; doi:10.1186/s12887-025-06242-y)
Supplement: Supplementary file 1 — Supplementary Material 1. [file 12887_2025_6242_MOESM1_ESM.docx]

Supplementary Information

Table S.1 Detailed cohort information. IFT: Impedance Field Telemetry. CT: Computed Tomography.

| ID Gender Etiology of hearing loss Side Array type Age in IFT  years^1^ type | | | | | | | CT/X-ray: days since IFT | Ground truth insertion depth (mm) | Predicted insertion depth (mm) |
| --- | --- | --- | --- | --- | --- | --- | --- | --- | --- |
| S01 female unknown (family history) left FLEX^soft^ 1 | | | | | | intraop | 1 | 2.3 | 2.1 |
| right FLEX^soft^ 1 | | | | | | intraop | 1 | -1.4 | -0.5 |
| S02 | male | genetic (Connexin-26 mutation) | left | Standard | 1 | intraop | 1 | 1.2 | 1.4 |
| S03 | male | unknown | left | FLEX^28^ | 1 | intraop | 0 | 0.6 | 0.6 |
|  |  |  | right | FLEX^28^ | 1 | intraop | 0 | 1.4 | -0.9 |
| S04 | female | Aymé-Gripp | left | Standard | 0 | postop | -33 | 2.3 | 2.4 |
|  |  |  | left^2^ | Standard | 3 | postop | -19 | -5.0 | -2.1 |
|  |  |  | right | Standard | 1 | postop | -38 | 2.5 | 2.0 |
| S05 | male | unknown | left | Standard | 1 | intraop | 1 | 2.4 | 1.2 |
| S06 | male | unknown | left | Standard | 2 | intraop | 1 | 1.7 | 0.3 |
| S07 | male | unknown | left | Standard | 1 | intraop | 1 | 1.0 | 2.6 |
|  |  |  | right | Standard | 2 | intraop | 3 | 2.3 | 1.6 |
| S08 | male | Waardenburg | right | Standard | 1 | intraop | 1 | 2.8 | 1.5 |
| S09 | female | unknown | left | Standard | 1 | intraop | 1 | 3.3 | 2.4 |
|  |  |  | right | Standard | 2 | intraop | 1 | 3.6 | 1.8 |
| S10 | female | unknown | right | FLEX^28^ | 6 | intraop | 1 | 2.8 | 2.4 |

S11 female unknown right FLEX^soft^ 2 intraop 3 1.2 1.1

| S12 | female | unknown (family history) | left | FLEX^28^ | 5 | postop | -24 | 3.5 | 2.6 |
| --- | --- | --- | --- | --- | --- | --- | --- | --- | --- |
|  |  |  | right | FLEX^28^ | 6 | postop | -35 | 5.0 | 2.0 |

S13 male Waardenburg left FLEX^soft^

| 2 | postop | -29 | 2.0 | 2.5 |
| --- | --- | --- | --- | --- |
| 1 | postop | -25 | 2.1 | 0.5 |

right FLEX^soft^

| S14 | male | unknown | left | FLEX^28^ | 5 | intraop | 1 | 1.2 | 2.8 |
| --- | --- | --- | --- | --- | --- | --- | --- | --- | --- |
| S15 male unknown left FLEX^soft^ 0 | | | | | | intraop | 1 | 1.4 | 2.6 |
| right FLEX^soft^ 0 | | | | | | intraop | 1 | 0.5 | 2.4 |

^1^Age at implantation.

^2^Revision surgery due to electrode migration.

| ID Gender Etiology of hearing loss Side Array type Age in IFT  years^1^ type | | | | | | | CT/X-ray: days since IFT | | Ground truth insertion depth (mm) | | Predicted insertion depth (mm) | |
| --- | --- | --- | --- | --- | --- | --- | --- | --- | --- | --- | --- | --- |
| S16 | male | unknown (premature birth) | left | Standard | 2 | intraop | | 1 | | 3.9 | | 1.6 |
|  |  |  | right | Standard | 2 | intraop | | 1 | | 2.3 | | 0.0 |
| S17 | male | unknown | right | FLEX^28^ | 6 | intraop | | 1 | | 2.7 | | 2.3 |
| S18 | male | genetic (GJB2 mutation) | left | Standard | 1 | intraop | | 1 | | 4.3 | | 2.6 |
|  |  |  | right | Standard | 0 | intraop | | 1 | | 2.5 | | 3.0 |
| S19 | male | unknown | left | Standard | 4 | intraop | | 1 | | 2.1 | | 1.9 |
|  |  |  | right | Standard | 4 | intraop | | 1 | | 2.9 | | 1.0 |
| S20 | female | unknown | left | FLEX^28^ | 1 | intraop | | 2 | | 1.8 | | 1.7 |
| S21 | male | unknown | left | FLEX^28^ | 1 | intraop | | 1 | | 3.2 | | 3.2 |
| S22 | female | unknown | left | Standard | 2 | intraop | | 3 | | 3.5 | | 2.0 |
| S23 | male | unknown | left | Standard | 2 | intraop | | 1 | | 0.0 | | 2.6 |
| S24 | male | unknown | right | Standard | 4 | intraop | | 1 | | 1.2 | | 1.8 |
| S25 | male | unknown | left | Standard | 1 | intraop | | 1 | | 3.1 | | 2.1 |
|  |  |  | right | Standard | 1 | intraop | | 1 | | 2.2 | | 1.9 |
| S26 | male | unknown | left | Standard | 2 | postop | | 29 | | 4.2 | | 1.8 |
| S27 | male | unknown (premature birth) | left | Standard | 1 | intraop | | 1 | | 2.9 | | 1.3 |
|  |  |  | right | Standard | 1 | intraop | | 1 | | 0.6 | | 1.7 |
| S28 | female | unknown | left | Standard | 0 | intraop | | 1 | | -6.2 | | -1.4 |
| S29 | male | unknown | right | FLEX^28^ | 6 | intraop | | 2 | | 2.1 | | 0.7 |
| S30 | female | CMV | left | FLEX^28^ | 1 | intraop | | 1 | | 2.4 | | 0.9 |
| S31 | male | meningitis | left | Standard | 2 | intraop | | 1 | | 2.8 | | 2.9 |
| S32 | male | unknown | left | Standard | 2 | intraop | | 1 | | 3.1 | | 2.2 |
|  |  |  | right | Standard | 2 | postop | | -36 | | 3.6 | | 2.4 |
| S33 | female | unknown (premature birth) | right | FLEX^28^ | 3 | intraop | | 0 | | 0.7 | | 0.6 |
| S34 | female | unknown | left | FLEX^28^ | 1 | intraop | | 1 | | 1.5 | | 2.5 |

^1^ Age at implantation.

| ID Gender Etiology of hearing loss Side Array type Age in IFT  years^1^ type | | | | | | | CT/X-ray: days since IFT | Ground truth insertion depth (mm) | Predicted insertion depth (mm) |
| --- | --- | --- | --- | --- | --- | --- | --- | --- | --- |
|  |  |  | right | FLEX^28^ | 1 | intraop | 1 | 3.6 | 2.6 |
| S35 | male | unknown | left | Standard | 4 | intraop | 1 | 0.6 | -0.2 |
| S36 | male | unknown | right | Standard | 3 | intraop | 1 | 2.8 | 1.3 |
| S37 | female | unknown | left | FLEX^28^ | 1 | intraop | 5 | 3.5 | 2.3 |

FLEXsoft FLEXsoft FLEXsoft

| S40 | female | unknown | left | FLEX^28^ | 1 | intraop | 0 | 1.2 | -1.3 |
| --- | --- | --- | --- | --- | --- | --- | --- | --- | --- |
| S41 | male | unknown | left | FLEX^28^ | 5 | intraop | 1 | 2.2 | 2.1 |
|  |  |  | right | Standard | 2 | intraop | 3 | 2.8 | 1.2 |

| 2 | intraop | 3 | 5.1 | 2.4 |
| --- | --- | --- | --- | --- |
| 2 | intraop | 3 | 1.4 | 2.1 |
| 6 | postop | -33 | 2.7 | 2.2 |

| S38 | male | unknown | left |
| --- | --- | --- | --- |
|  |  |  | right |
| S39 | female | unknown | right |

^1^Age at implantation.
